# Reversible Pulmonary Hypertension Related to Hyperthyroidism: A Case Report

**DOI:** 10.7759/cureus.51377

**Published:** 2023-12-31

**Authors:** Joana Silva, Ana Carvoeiro, Paula Cerqueira, André Calheiros, Carlos Gonçalves

**Affiliations:** 1 Internal Medicine, ULSAM (Unidade Local de Saúde do Alto Minho) Hospital Conde de Bertiandos, Ponte de Lima, PRT; 2 Internal Medicine, ULSAM (Unidade Local de Saúde do Alto Minho) Hospital de Santa Luzia, Viana do Castelo, PRT

**Keywords:** tricuspid regurgitation, right cardiac catheterization, right ventricular dysfunction, reversible pulmonary hypertension, graves disease, hyperthyroidism

## Abstract

A systematic assessment is crucial to confirm the diagnosis of pulmonary hypertension (PH) and classify it based on its etiological mechanism. This case report describes a young woman with a recent diagnosis of Graves’ disease who presented with exertional dyspnea and fatigue. The initial ultrasound heart examination indicated moderate tricuspid regurgitation, an increased estimated systolic pulmonary artery pressure (sPAP), and suggestive alterations of atrial septal communication. For a more detailed characterization of this aspect, a transesophageal echocardiogram (TEE) was performed, which confirmed, through the agitated saline injection method, the presence of a patent foramen ovale (PFO). Further investigation for common causes of pulmonary hypertension yielded negative results. Treatment with methimazole and radioiodine ablation with glucocorticoid coverage was made. One year later, the patient reached a euthyroid state and reported an improvement in the symptoms. Follow-up transthoracic echocardiogram revealed resolution of pulmonary hypertension, with a normal sPAP and normal-sized right chambers. Right heart catheterization confirmed the normal findings.

Hyperthyroidism is considered a potential cause of pulmonary hypertension through the effects of high cardiac output and autoimmune-induced pulmonary vascular endothelium injury. As such, it should be included in the etiological investigation of suspected pulmonary hypertension, as its cardiovascular manifestations may be completely reversible without the need for targeted therapy.

## Introduction

The cardiovascular manifestations of hyperthyroidism have been documented in the literature since at least 1980 [1], but some aspects remain unclear. Excess thyroid hormone states correlate with an increased basal metabolic rate, accompanied by an increased total blood volume, reduced systemic vascular resistance, positive inotropic and chronotropic effects, and shortened circulation time [2]. Considering that the right heart is more susceptible to volume overload and a hyperdynamic circulatory state, hyperthyroidism can rapidly lead to right ventricular dysfunction, due to compromised functional reserve, and leading to consequent symptoms of heart failure [3].

An isolated abnormal rise in mean pulmonary artery pressure is insufficient by itself to define pulmonary vascular disease. This increase can be attributed to other factors, such as increased cardiac output, pulmonary artery wedge pressure, or volume overload, as seen in liver disease, lung disease, or hyperthyroidism [4]. According to the hemodynamic definition, pulmonary hypertension (PH) is defined by mean pulmonary arterial pressure (mPAP) >20 mmHg at rest assessed by right heart catheterization [4,5]. The arbitrariness of the PH definition has been overcome, as the new definition was grounded in comprehensive studies that assess the physiological thresholds of systolic pulmonary artery pressure in healthy individuals, as well as cutoff values with prognostic significance [6].

Case reports of PH, tricuspid regurgitation, and right-sided heart failure associated with hyperthyroidism have been described in the literature [7-11]. Therefore, in subjects with elevated pulmonary artery pressure, an exhaustive assessment should be carried out to confirm the diagnosis and identify any potential reversible underlying cause.

This case was previously presented as a meeting abstract at the 2022 ECIM European Congress of Internal Medicine on June 9, 2022.

## Case presentation

A 33-year-old woman, recently diagnosed with Graves disease and treated with methimazole and propranolol for six months, presented with progressively worsening exertional dyspnea, orthopnea, and fatigue over the last few weeks. She reported no peripheral edema, palpitations, chest pain, or syncope on exertion. There was no history of illicit drug use, medical treatment, cigarette smoking, or sleep apnea, and no family history of pulmonary hypertension.

On physical examination, the patient was alert, aware of her surroundings, and able to interact appropriately. The skin and mucous membranes had a normal color, and the patient was adequately hydrated. She showed no signs of respiratory distress in room air, with a peripheral oxygen saturation of 98%. Additionally, there was no observed decrease in saturation during exercise. Positive jugular venous turgor was noted, and cardiac auscultation revealed rhythmic sounds with an early systolic murmur of Grade II/III intensity detected at the aortic focus. Pulmonary auscultation indicated the absence of bilateral crackles, and there was no evidence of hepatomegaly or peripheral edema.

The laboratory results unveiled the absence of anemia, denoted by a hemoglobin level of 12.3 g/dL. White blood cell and platelet counts stood within the expected normal ranges (7.670/µL and 222.000/µL, respectively). Additionally, key parameters, including erythrocyte sedimentation rate (8 mm/h), serum creatinine (0.63 mg/dL), and liver parameters, including alanine aminotransferase, aspartate aminotransferase, alkaline phosphatase, gamma-glutamyl transferase, bilirubin, albumin, total protein, prothrombin time, and international normalized ratio (INR), displayed values that conformed to established normal ranges. The B-type natriuretic peptide assay yielded a negative result, registering at 12 pg/mL. Thyroid function assessments indicated a Thyroid Stimulating Hormone (TSH) level of 0.000 IU/mL, a free T4 level of 2.10 ng/dL, and a free T3 level of 9.06 pg/mL. HIV, hepatitis B, and hepatitis C serology were nonreactive. Thyroid peroxidase antibodies were positive (>1000 UI/mL) and the remaining immunologic panel was negative.

**Figure 1:**
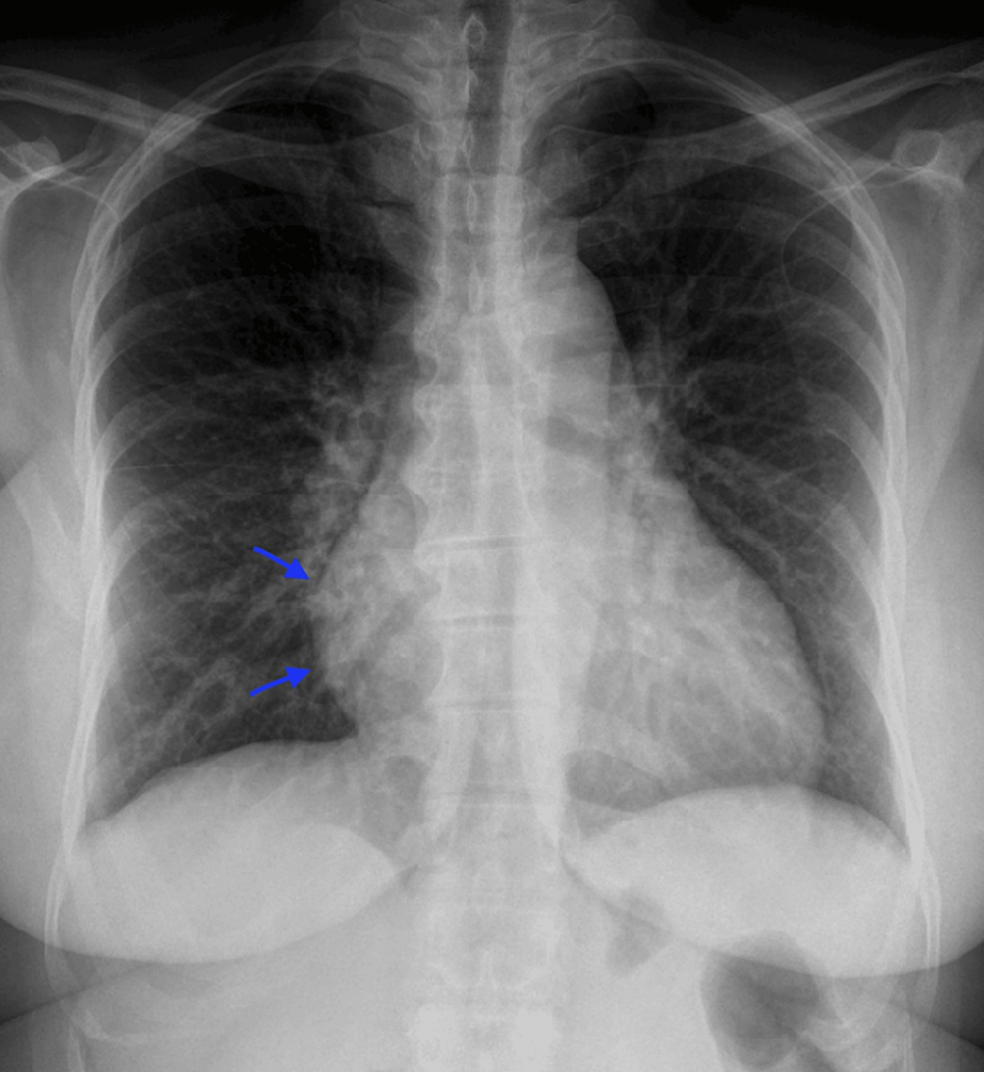
Chest X-ray at initial investigation Chest X-ray reveals mild enlargement of the cardiac silhouette (arrows)

The 12-lead electrocardiogram showed a sinus rhythm with well-defined P waves, a normal PR interval, and a standard QRS complex duration, and the ST segment and T waves exhibited no abnormalities. The calculated heart rate was 90 beats per minute, and there was no significant axis deviation. Chest X-ray revealed a mild enlargement of the cardiac silhouette (Figure 1), without signs of pulmonary congestion.

Transthoracic echocardiogram (TTE) findings revealed mild to moderate dilation of the left atrium and slight dilation of the right atrium. The ventricles demonstrated dimensions within normal limits, and the walls exhibited normal thickness. A color Doppler flow pattern indicative of an atrial septal defect was visualized, pending further characterization through a transesophageal echocardiogram. The interventricular septum appeared intact without evidence of flattening. Moderate tricuspid regurgitation was observed, and the estimated sPAP measured at 54 + 5 mmHg. The inferior vena cava was not dilated, with respiratory variability exceeding 50%. Biventricular systolic function was preserved, and there were no discernible alterations in segmental contractility at rest. Notably, there was an absence of pericardial effusion. TEE documented the presence of a patent foramen ovale (PFO) measuring 0.6x1.2 mm, along with a non-significant left-to-right shunt, using the agitated saline injection method.

**Figure 2:**
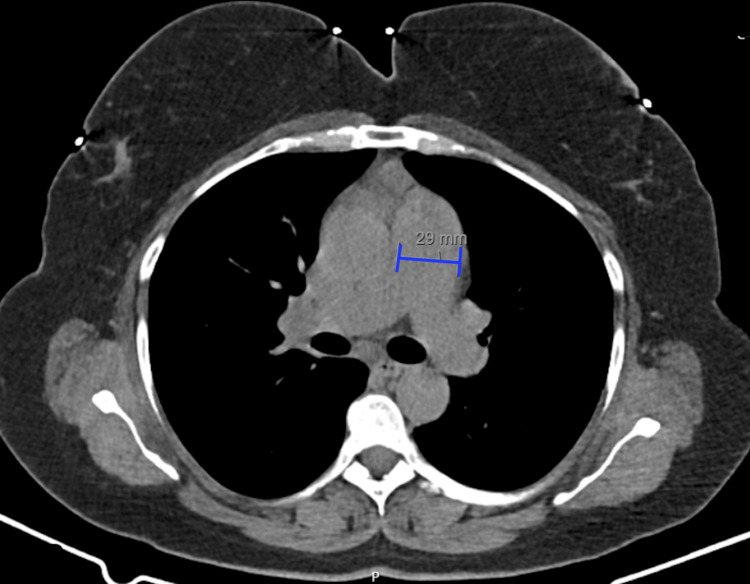
Computed tomography pulmonary angiography (CTPA) CTPA reveals a pulmonary artery caliber at the upper limit of normal (measuring 29 mm)

The pulmonary function tests were normal, with no indicative alterations suggestive of pulmonary disease. The computed tomography pulmonary angiography (CTPA) revealed a pulmonary artery caliber measured at 29 mm, which is at the upper limit of normal (Figure 2). CTPA results ruled out pulmonary thromboembolism, confirming a normal pulmonary vasculature.

Faced with the diagnosis of pulmonary hypertension, without right ventricular dysfunction, and uncontrolled hyperthyroidism, the patient underwent radioiodine ablation with glucocorticoid coverage. Six months later, upon reevaluation through echocardiography, mild dilation of the right atrium was documented, with the remaining cardiac cavities within normal dimensions, and an improvement in estimated systolic pulmonary artery pressure (32 + 5 mmHg).

One year later, the patient achieved euthyroid status and reported a significant improvement in symptoms. Subsequent follow-up TTE revealed a normal estimated systolic pulmonary artery pressure (Table 1) and normal-sized chambers. Right heart catheterization findings confirmed an mPAP below 20 mmHg, along with normal intracardiac pressures and a Qp/Qs ratio of 1. 

**Table 1:** Evolution of thyroid function, sPAP, and tricuspid regurgitation in the patient under hyperthyroidism treatment TSH – Thyroid stimulating hormone (normal range: 0.35 – 4.94 UI/mL); FT4 – free T4 hormone (normal range: 0.70 – 1.48 ng/dL); FT3 – free T3 hormone (normal range: 1.71 – 3.71 ng/dL); sPAP – systolic pulmonary arterial pressure; TR – tricuspid regurgitation

	At presentation	6 months later	12 months later
TSH (UI/mL)	< 0.001	0.28	3.2
FT4 (ng/dL)	2.10	1.64	0.80
FT3 (pg/mL)	9.06	4.27	1.88
sPAP (mmHg)	54 + 5	32 + 5	21 + 5
TR	moderate	mild	mild

The patient is currently under routine follow-up, and there has been no recurrence of dyspnea or other clinical manifestations.

## Discussion

Many of the clinical manifestations of hyperthyroidism result from the ability of thyroid hormones to influence cardiovascular hemodynamics [12]. Possible mechanisms for the development of pulmonary hypertension due to hyperthyroidism include damage to the pulmonary vascular endothelium due to high cardiac output, autoimmune process, increased metabolism of intrinsic pulmonary vasodilators, and vascular vasoconstriction due to decreased cholinergic output [2,13].

In this case, pulmonary hypertension initially seemed to have two fundamental etiologies, with possible contributions from both a PFO and presumably hyperthyroidism. The initial transthoracic echocardiogram showed, in addition to an increased systolic pulmonary artery pressure, suggestive alterations of atrial septal communication. For a more detailed characterization of this aspect, a transesophageal echocardiogram was performed, documenting the presence of a patent foramen ovale with a non-significant left-to-right shunt observed through the agitated saline injection method. The purpose of the transesophageal echocardiogram (TEE) was to determine whether the findings in the transthoracic echocardiogram indicated a congenital heart defect with hemodynamic consequences on the right heart, potentially contributing to pulmonary hypertension. While the presence of a PFO was confirmed on TEE, its sole association with pulmonary hypertension is insufficient.

Recognizing that performing right heart catheterization earlier could have resulted in a more precise diagnosis and characterization of pulmonary hypertension, the strategy of managing hyperthyroidism before undergoing this invasive procedure seemed essential in the management of this case. Subsequently, right heart catheterization, performed when the patient was in a euthyroid state, revealed normal results, particularly showing a Qp/Qs ratio of 1, suggesting no significant shunting or abnormal blood flow within the heart. This indicates a complete reversal of systolic pulmonary artery pressure with the ongoing management of hyperthyroidism and the absence of contribution from the PFO for PH. In fact, in this case, the authors believe that the initially increased estimated systolic pulmonary arterial pressure on the first echocardiography was associated with uncontrolled hyperthyroidism.

Despite not being observed in this patient, it is common for individuals diagnosed with hyperthyroidism to also have concurrent atrial fibrillation. Therefore, a strategy of heart rate control with beta-blockers is recommended, as these are also one of the cornerstones of the treatment of hyperthyroidism.

Fortunately, the patient did not develop right ventricular dysfunction, and the pulmonary hypertension was reversed with adequate control of thyroid function, emphasizing the favorable prognosis noted by several authors in similar cases. Notably, when hyperthyroidism results in right ventricular dysfunction, this can lead to heart failure, associated complications, and an increased risk of mortality.

## Conclusions

Hyperthyroidism appears to be a potential cause of pulmonary hypertension. Consequently, it should be part of the etiological investigation of suspected or diagnosed pulmonary hypertension. This consideration is necessary due to the treatable nature of hyperthyroidism. Pulmonary hypertension, as observed in this case report, has the potential for complete reversibility through proper treatment and effective control of increased thyroid activity.

Although cardiovascular symptoms are considered a part of thyrotoxicosis, patients recently diagnosed with or newly presenting symptoms of Graves' disease should be questioned about the presence of symptoms such as dyspnea on exertion, orthopnea, fatigue, and syncope during or shortly after physical exertion. Those who are symptomatic warrant further assessment through echocardiography to identify potential signs suggestive of pulmonary hypertension. This proactive approach ensures a comprehensive evaluation and facilitates ongoing monitoring and management of the cardiovascular consequences of hyperthyroidism.
